# Acute ischemic stroke prediction and predictive factors analysis using hematological indicators in elderly hypertensives post-transient ischemic attack

**DOI:** 10.1038/s41598-024-51402-2

**Published:** 2024-01-06

**Authors:** Chang Shu, Chenguang Zheng, Da Luo, Jie Song, Zhengyi Jiang, Le Ge

**Affiliations:** 1 Tianjin Key Laboratory of Cerebral Vascular and Neurodegenerative Diseases, Tianjin Neurosurgical Institute, Tianjin Huanhu Hospital, Tianjin, 300350 China; 2 Tianjin Key Laboratory of Brain Science and Neural Engineering, Tianjin University, Tianjin, China; 3 Academy of Medical Engineering and Translational Medicine, Intelligent Medical Engineering, Tianjin University, Tianjin, China

**Keywords:** Risk factors, Cerebrovascular disorders, Stroke, Data mining, Machine learning

## Abstract

Elderly hypertensive patients diagnosed with transient ischemic attack (TIA) are at a heightened risk for developing acute ischemic stroke (AIS). This underscores the critical need for effective risk prediction and identification of predictive factors. In our study, we utilized patient data from peripheral blood tests and clinical profiles within hospital information systems. These patients were followed for a three-year period to document incident AIS. Our cohort of 11,056 individuals was randomly divided into training, validation, and testing sets in a 5:2:3 ratio. We developed an XGBoost model, developed using selected indicators, provides an effective and non-invasive method for predicting the risk of AIS in elderly hypertensive patients diagnosed with TIA. Impressively, this model achieved a balanced accuracy of 0.9022, a recall of 0.8688, and a PR-AUC of 0.9315. Notably, our model effectively encapsulates essential data variations involving mixed nonlinear interactions, providing competitive performance against more complex models that incorporate a wider range of variables. Further, we conducted an in-depth analysis of the importance and sensitivity of each selected indicator and their interactions. This research equips clinicians with the necessary tools for more precise identification of high-risk individuals, thereby paving the way for more effective stroke prevention and management strategies.

## Introduction

Transient ischemic attack (TIA) and acute ischemic stroke (AIS) are both characterized by a sudden reduction in blood flow, leading to temporary or permanent loss of neurological function^1^. TIA is defined as a transient episode of neurologic dysfunction due to focal brain, spinal cord, or retinal ischemia, without acute infarction^2^. Recent studies have shown a strong correlation between TIA and the subsequent development of AIS^3,4^. Approximately 20% of TIA patients experience an AIS within three months of the initial TIA event, with the highest risk occurring within the first 48 h^5^. Over the long term, TIA patients face a 10-year stroke risk of 19% and a combined 10-year risk of stroke, myocardial infarction, and vascular death at 43%^6^. TIA and AIS share several common risk factors, such as hypertension, diabetes mellitus, hyperlipidemia, and atrial fibrillation^7^. Among these, hypertension is the most prevalent risk factor for both conditions^8^. Elderly patients with hypertension who experience TIA symptoms, such as sudden weakness or numbness in the face, arms, or legs; confusion; difficulty speaking; vision problems; dizziness; and severe headache, are at an increased risk of developing AIS in the days and weeks following the TIA event^9^. The unpredictability of progression from TIA to AIS not only imposes a considerable burden on the healthcare system but also significantly impacts the mental well-being and daily activities of elderly hypertensive patients. Given these risks and the urgent need to identify predictive factors, establishing an effective risk prediction model for AIS following a TIA event in elderly hypertensive patients is crucial. However, the current literature on AIS prediction predominantly focuses on broader patient populations^10–12^, often overlooking the unique characteristics and risk profiles of this specific group.

A peripheral routine blood test (RBT) is the most commonly performed clinical test and provides a comprehensive evaluation of various blood components and characteristics. This evaluation offers valuable insights into an individual's hematological profile, reflecting their overall health status^13,14^. The direct measurements obtained from the RBT are known as primary hematological indicators (PHIs). Additionally, derived hematological indicators (DHIs), which are calculated from PHIs using various mathematical methods, are included. Together, these indicators are collectively referred to as primary and derived hematological indicators (PDHIs). Supplementary Material 1 in this study provides a comprehensive list of all PDHIs measured using the Sysmex XE 5000 Hematology Analyzer, including their full names, corresponding abbreviations, and the methodologies for calculating the DHIs. Numerous studies have indicated the critical role of PDHIs in the development and progression of hypertension, TIA, and AIS^15,16^. Moreover, there is substantial evidence of common alterations in PDHIs across these three vascular-origin diseases. For instance, an elevation in neutrophils and a decrease in lymphocytes^17–19^, as well as consistent changes in hematocrit^20–22^ and red cell distribution width (RDW)^14,23,24^, have been observed across different studies focusing on these three vascular-origin diseases. These findings suggest the presence of numerous shared hematological indicators within the internal environment of patients with these vascular diseases. These shared hematological indicators may hold the key to predicting the risk of AIS in elderly patients with hypertension who have experienced a TIA.

Despite the recognized importance of these PDHIs, a comprehensive and systematic study investigating their predictive power and associated risk factors for AIS following a TIA in elderly hypertensive patients is lacking. The complexities inherent in PDHIs, such as nonlinear relationships^25^ and multicollinearity^26^, necessitate the use of advanced data science methodologies to unlock their predictive potential and unravel associated risk factors. To address these challenges, this paper employs a robust analytic strategy by initially utilizing the searching for uncorrelated list of variables (SULOV)-recursive method, tailored for nonlinear data to select relevant variables while minimizing redundancy. Subsequently, an extreme gradient boosting (XGBoost) model, known for its efficacy in handling multicollinearity and capturing complex interaction relationships, is constructed. The model is fine-tuned through an exhaustive hyperparameter optimization process and further calibrated to enhance predictive accuracy. This comprehensive approach aims to construct a reliable three-year AIS risk prediction model for elderly hypertensive TIA patients, harnessing the full spectrum of PDHIs. The model's interpretability and sensitivity analysis are designed to identify and highlight the key factors that contribute to the progression from TIA to AIS in this high-risk group. Our research aims to enhance early prediction and intervention for AIS, potentially improving management and outcomes for elderly hypertensive patients post-TIA.

## Methods

### Cohort selection and variables definition

Our study extracted data from the Hospital Information System (HIS) and included 32,643 elderly patients consecutively admitted with a history of hypertension and subsequently received a primary diagnosis of TIA at Tianjin Huanhu Hospital's emergency department from July 2015 to December 2019. Follow-ups were conducted using a bulk mobile messaging-WeChat-remote follow-up system, supplemented by phone calls when necessary, to determine if the patients experienced cerebral infarction within three years post-TIA. Outcomes were gauged using a binary question: "Have you, or the patient, been diagnosed with cerebral infarction, confirmed by a neurologist's cranial CT or MRI scan, within three years following the TIA diagnosis at our hospital?" If direct contact failed, we reached out to relatives based on contact information provided during the initial hospital visit. This methodology ensured precise identification of AIS incidents within the follow-up window. We excluded patients based on the following criteria: (1) Patients were diagnosed with chronic cerebral infarction or other cerebrovascular diseases based on cranial CT or MRI scan reports in the EMR system. (2) Patients admitted to the emergency department who did not have a completed electronic medical record, thus precluding the extraction of RBT report, past medical history, and alcohol and tobacco use data. (3) Patients whose admission blood routine tests showed white blood cell (WBC), red blood cell (RBC), or platelet counts (PLT) outside of the normal range (WBC: 4–10 × 10^9^ cells/L, RBC: 4–6 × 10^12^ cells/L, PLT: 150–450 × 10^9^ cells/L), were excluded. This criterion was set to mitigate the impact of infections and hematological diseases on the PDHIs. (4) Patients admitted to the emergency department who did not provide contact information. (5) Patients lost to follow-up. Supplemental Fig. 1 is the flowchart of patient selection. Our research incorporated 28 PHIs, eight DHIs, and eight categorized demographic and lifestyle variables including gender, age (categorized into three groups: 60–69, 70–79, and 80 + years), drinking history, and past medical history. All PDHIs (n = 36) used in this study are continuous variables, and they were obtained from the first blood draw post-admission. A comprehensive list of these PDHIs is provided in Supplementary Material 1.

**Figure 1:**
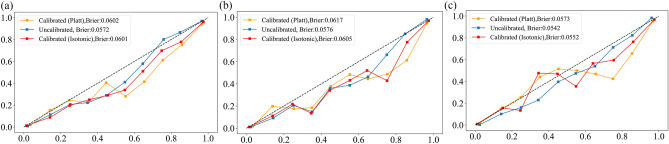
Model calibration. The results of model calibration for XGB-PDHIs model (**a**), XGB-Mixed model (**b**) and XGB-All model (**c**).

All data used in this study were extracted from the hospital's business system with risk minimization measures to ensure data security. Private information, such as patient names, ID numbers, and addresses, was hidden, and data usage was in compliance with the provisions for informed exemption of the hospital ethics committee. Informed consent was obtained from all subjects or their legal guardian(s). The study was approved by the Huanhu Ethics Committee (No. 2021060). This study has been registered in the Chinese Clinical Trial Registry (https://www.chictr.org.cn/login.aspx?referurl=%2flistbycreater.aspx), with the registration number ChiCTR2100054189. All methods were performed in accordance with the Helsinki Declaration for human research.

### Handling collinearity and variable selection

Considering the significant correlations among PDHIs internally, we employed the SULOV-Recursive method^27^. The SULOV (Searching for Uncorrelated List of Variables) algorithm is an adaptation of the Minimum-Redundancy-Maximum-Relevance (MRMR) method, designed to handle multicollinearity. It works by identifying pairs of highly correlated variables, assessing their relevance to the target using the Mutual Information Score, and subsequently excluding the less informative variable of each pair. This process leaves a set of variables with maximum informational value and minimal mutual correlation. Subsequently, the algorithm deploys the XGBoost machine learning method in an iterative manner to pinpoint the most predictive variables, conducting multiple training-validation cycles and collating top features from varying data subsets. This procedure concludes by discarding redundant variables, providing a streamlined and effective set of predictors for the subsequent modeling stages. For the demographic and lifestyle variables, we applied integer encoding. The Cramer’s V correlation matrix algorithm was utilized to further remove multicollinearity among these categorical variables. For both continuous and categorical variables, we set the correlation threshold at 0.3.

### Non-linearity assessment and modeling workflow

To assess the potential non-linearity between the final selected PDHIs and the outcome variable, we employed the Box-Tidwell test. This test investigates the linearity of predictors with respect to the logit of the outcome variable by introducing log-transformed interaction terms between the continuous predictors and their respective natural logs. This step is crucial as it aids us in making an informed choice about the appropriate predictive model to employ. A significant interaction term (p ≤ 0.05) signifies the presence of non-linearity.

Supplemental Fig. 2 illustrates the overall workflow of our model fitting and testing. To address the combined linear and non-linear characteristics of our data, we employed XGBoost as our principal model. This choice was based not only on the preliminary screening results from our training and validation sets, which demonstrated XGBoost's superior performance among 15 different machine learning algorithms, but also on its considerable suitability for handling medical tabular data, as evidenced by the relevant literature in the field^28–30^. Three different XGBoost models were constructed. The first model utilized only the selected PDHIs as input. The second model incorporated both the selected PDHIs and categorical variables, while the third included all variables without feature selection. After applying Robust Scaler for continuous variables and Label Encoding for categorical variables, we tuned the hyperparameters for each XGBoost model using the Tree-structured Parzen Estimator (TPE) method within the Optuna framework^28^. During the model training phase, we integrated a ten-fold cross-validation process. For each fold, class imbalance was addressed uniquely for each of the three models: applying the Synthetic Minority Over-sampling Technique (SMOTE) to the training subset for the model with only PDHIs^31^, and SMOTENC for the models including both PDHIs and categorical variables^32^. This treatment was restricted to the nine out of ten folds used for training in each cross-validation iteration. The remaining one fold, serving as the validation set, was kept untouched by either SMOTE or SMOTENC, thus preserving its original distribution. After hyperparameter tuning, we performed model calibration on the initially separated validation set, utilizing isotonic regression and sigmoid calibration methods. The optimal calibration approach for each model was determined by comparing the uncalibrated model with these two methods, selecting the one that yielded the lowest Brier score. To evaluate the performance of the three calibrated models, we employed a ten-fold cross-validation approach on the training set, incorporating appropriate class imbalance adjustments. This enhanced the models' ability to detect minority classes and ensured a balanced performance evaluation, preventing the overestimation of accuracy due to imbalanced class distributions. For the ultimate evaluation on the test set, we abstained from applying class imbalance processing to prevent data leakage and to ensure that the models' performance reflected a more realistic prediction scenario, where the original class distribution was maintained. In our study, three calibrated XGBoost models with varying input variables were developed in parallel. Each model underwent a rigorous process of hyperparameter tuning using cross-validation on the training set and calibration on an independent validation set. To assess the performance of these models, we initially conducted a comparative analysis using McNemar's test with Benjamini–Hochberg correction^33,34^, applying it to both the validation and test sets. This is a statistical method used for comparing the predictive capabilities of already fitted classifiers.

**Figure 2:**
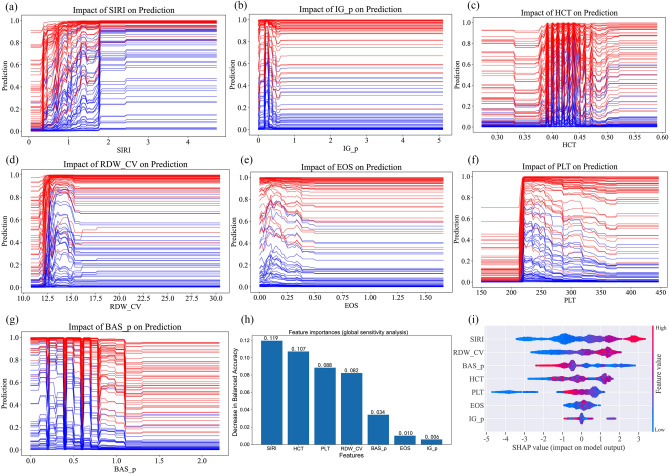
Risk factor analysis. Individual sensitivity analysis for SIRI (**a**), IG_p (b), HCT (**c**), RDW_CV (**d**), EOS (**e**), PLT (**f**), and BAS_P (**g**). Global sensitivity analysis for these predictive factors (**h**). Global SHAP value plot demonstrating the overall effect ranking (**i**).

### Multi-tiered approach for predictive factor analysis

In this study, we adopted a multi-tiered approach for our predictive factor analysis. In the individual sensitivity analysis, we systematically varied the value of each selected PDHIs within its observed range, evaluating how these changes influenced the model's predictions for specific patients. In the global sensitivity analysis, we randomly shuffled the values of each PDHI across the entire dataset, disrupting their original correlations with the target variable. This process enabled us to evaluate the independent contribution of each PDHI to the model's predictive performance. Following the sensitivity analyses, we applied the SHAP (SHapley Additive exPlanations) methodology to rank risk factors according to their importance^35^. The ranking is derived from each feature's SHAP value, which quantifies both the direct (main effect) and interaction contributions of each PDHI to the predictive outcome. The SHAP values essentially capture a feature's average contribution to the prediction outcome, considering all possible coalitions of features. Finally, we examined the interaction effects among the risk factors utilizing SHAP interaction values. This step uncovered the pairs of risk factors that significantly interact with each other, thereby shedding light on the complex interdependencies among the PDHIs.

### Sample size and statistical analysis

We performed a power analysis for the sample size determination of our training, validation, and test sets using the R Package 'pmsampsize'. This package computes the minimum sample size required for developing a multivariable prediction model. It specifies an anticipated AUC of 0.9 and utilizes the expected prevalence to approximate the Cox-Snell R-squared, following the methodology proposed by Riley et al.^36^. In our study, for the dataset with 44 input variables, the minimum sample size required is 989 cases. For the dataset with 14 input variables, it is 315 cases, and for the dataset with 7 input variables, it is 303 cases. The sizes of our training, validation, and test sets significantly exceed these thresholds, indicating a reduced risk of overfitting and ensuring precise estimation of key parameters in the prediction models. This substantial sample size provides a robust foundation for the development and validation of our models.

Continuous variables were reported as medians with interquartile range (IQR) and categorical variables as percentages. Statistical comparisons were performed using the Kruskal‒Wallis and chi-squared tests. P ≤ 0.05 for statistical significance. In this study, given the characteristics of imbalanced data and our practical experience, balanced accuracy was employed as the primary optimization metric to rank the performance of these models. In our model evaluation, we also reported other metrics. For detailed introductions to these metrics, please refer to Supplementary Material 2. The computer program was implemented in Python 3.8.13, with XGBoost (1.6.1), scikit-learn (1.1.1), SHAP (0.41.0), running on Ubuntu 20.04.

## Results

### Data split, variables selection and nonlinear detection

Our study cohort consisted of 11,056 elderly patients diagnosed with TIA, having a mean age of 68 [@@64, 73] and a male to female ratio of 5451:5605. All patients had a history of hypertension. By applying a random shuffle strategy, the cohort was randomly split into training (n = 5527), validation (n = 2212), and testing datasets (n = 3317) at a ratio of 5:2:3. The proportions of positive outcomes were 28.2% in the training set, 26.8% in the validation set, and 27.8% in the test set. The descriptive statistics of variables across these datasets are provided in Supplementary Table 1. A pairwise Pearson correlation analysis was performed on 36 PDHIs in the training set (Supplementary Table 2). We found 12 pairs of PDHIs with absolute correlation coefficients greater than 0.9, and 48 pairs with coefficients greater than 0.7, indicating multicollinearity among the PDHIs data. the application of the SULOV algorithm effectively reduced multicollinearity among PDHIs, identifying seven key indicators (SIRI, HCT, RDW_CV, PLT, IG_p, BAS_p and EOS) with mutual correlation coefficients below 0.3. Similarly, using the Cramer’s V correlation matrix for categorical variables, we pinpointed seven significant factors: smoking status, alcohol consumption, diabetes, heart disease, respiratory disorders, gender, and age, each exhibiting a correlation coefficient under 0.3.

Based on the results of the Box-Tidwell test, we observed that in the training set, the predictors 'HCT', 'RDW_CV', 'PLT', and 'SIRI' showed p-values less than 0.05, indicating non-linear relationships with the outcome. Conversely, 'IG_p' (p = 0.168), 'BAS_p' (p = 0.413), and 'EOS' (p = 0.375) had p-values greater than 0.05, suggesting linear relationships. Given the presence of both linear and non-linear relationships among the variables, we opted for the versatile XGBoost algorithm for our modeling, following an initial screening of 15 machine learning algorithms (Supplemental Fig. 3).

**Figure 3:**
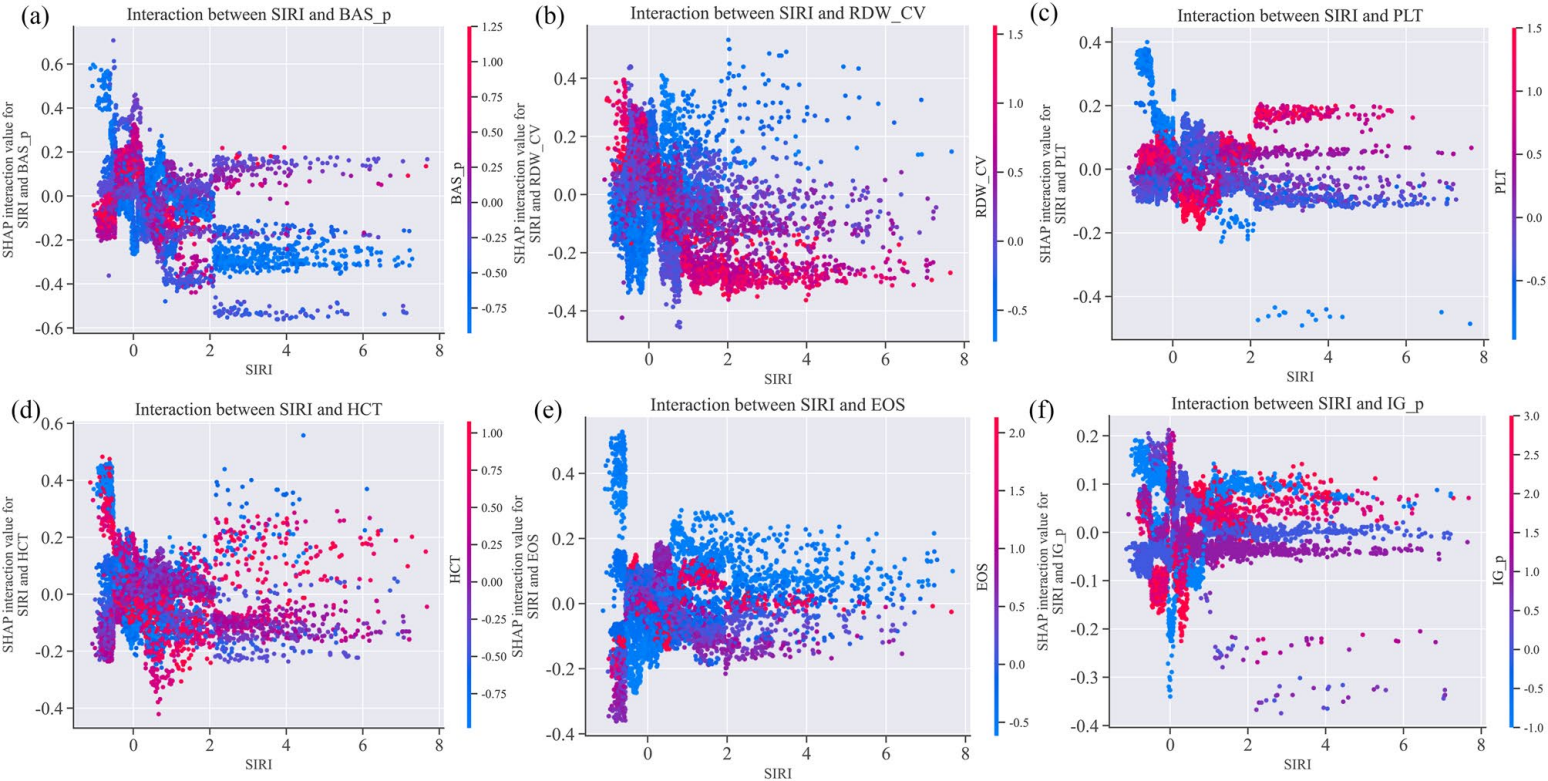
SHAP interaction values plots. Utilizing SHAP interaction values, we visualized the interactive effects between SIRI and other predictive factors. The x-axis represents the values of SIRI after robust scaling. The color gradient in the plot, from green to red, indicates the increasing values of other predictive factors (**a** BAS_p, **b** RDW_CV, **c** PLT, **d** HCT, **e** EOS, **f** IG_p) post robust scaling. The y-axis shows the calculated SHAP interaction values between SIRI and these predictive factors, reflecting the impact of their interactions on the model's prediction for each sample.

### Model fitting and performance evaluation

We employed three models for thorough assessment of variable fitting to the outcome. These included XGBoost with only selected PDHIs (XGB-PDHIs); XGBoost featuring both selected PDHIs and categorical variables (XGB-Mixed); XGBoost incorporating all variables without feature selection (XGB-All). The optimal hyperparameters determined for each model after tuning are outlined in Supplementary Table 3. The probability calibration results are depicted in Fig. 1. It was observed that for the three XGBoost models, the Brier scores were higher after calibration. Hence, the uncalibrated versions of these models were selected for further dataset evaluation. Table 1 outlines the results of our assessment, featuring the performance metrics of the optimized models as evaluated through tenfold cross-validation on the training set, and their ultimate evaluation on the test set. The slightly lower metrics on the test set, in comparison to the training set cross-validation results, indicate that our model maintains good generalization capabilities. This finding suggests that our model has effectively learned the underlying patterns in the data without overfitting to the training set, thereby ensuring its applicability to real-world scenarios.

**Table 1:** Model performance assessment through cross-validation on training set and independent evaluation on test set.

Metrics	Cross-validation on training set			Independent evaluation on test set		
	XGB-PDHIs	XGB-Mixed	XGB-All	XGB-PDHIs	XGB-Mixed	XGB-All
Bal-ACC	0.9082 ± 0.0212	0.9101 ± 0.0167	0.9122 ± 0.0182	0.9022	0.9031	0.9077
ROC-AUC	0.9713 ± 0.0069	0.9715 ± 0.0056	0.9732 ± 0.0067	0.9700	0.9721	0.9735
PR-AUC	0.9350 ± 0.0132	0.9347 ± 0.0112	0.9396 ± 0.0126	0.9315	0.9362	0.9379
Recall	0.8837 ± 0.0359	0.8921 ± 0.0299	0.8908 ± 0.0309	0.8688	0.8742	0.8764
Precision	0.8377 ± 0.0265	0.8304 ± 0.0272	0.8407 ± 0.0290	0.8387	0.8318	0.8470
Specificity	0.9327 ± 0.0116	0.9282 ± 0.0130	0.9335 ± 0.0133	0.9357	0.9319	0.9390
F1-score	0.8598 ± 0.0278	0.8598 ± 0.0221	0.8647 ± 0.0245	0.8535	0.8525	0.8614
C-Kappa	0.8029 ± 0.0387	0.8020 ± 0.0312	0.8095 ± 0.0346	0.7957	0.7937	0.8068
F2-score	0.8740 ± 0.0319	0.8788 ± 0.0252	0.8801 ± 0.0271	0.8626	0.8654	0.8703
Jaccard	0.7552 ± 0.0427	0.7547 ± 0.0343	0.7625 ± 0.0384	0.7444	0.7429	0.7566
MCC	0.8037 ± 0.0390	0.8034 ± 0.0315	0.8105 ± 0.0348	0.7959	0.7942	0.8070

*Bal-ACC* balanced accuracy, *ROC-AUC* Area Under the Receiver Operating Characteristic Curve, *PR-AUC* Area Under the Precision-Recall Curve, *C-Kappa* Cohen's Kappa, *Jaccard* Jaccard Index, *MCC* Matthews Correlation Coefficient.

To compare the classification abilities of the three final fitted models, we employed McNemar's test in conjunction with the Benjamini-Hochberg (BH) correction. From our results (Table 2), we found no significant difference in the predictive capabilities of the three examined models on different data set, even though they each yielded different McNemar test statistics. This lack of statistical distinction suggests that, given our dataset, the predictive performances of the three models are effectively indistinguishable. Moreover, the input data for the XGB-PDHIs model consist solely of objectively measured continuous variables, which can be easily obtained through a single routine blood test, making it highly suitable for clinical application. Considering both its performance and simplicity, we chose the XGB-PDHIs model for in-depth interpretation.

**Table 2:** Table of McNemar's test results.

XGBoost model	Validation set				Test set			
	Statistic*	p-val	Adj.p-val	Reject H0*	Statistic	p-val	Adj.p-val	Reject H0
PDHIs versus Mixed	51	0.0487	0.1460	FALSE	82	0.8771	0.8771	FALSE
PDHIs versus All	66	0.1230	0.1845	FALSE	89	0.3852	0.6656	FALSE
Mixed versus All	51	0.8454	0.8454	FALSE	64	0.4437	0.6656	FALSE

PDHIs: XGB-PDHIs, Mixed: XGB-Mixed, All: XGB-All. Adj.p-val: the p-value adjusted for multiple testing using the Benjamini–Hochberg correction.

*Statistic refers to the number of discordant pairs from the contingency table used in McNemar's test. *H0 assumes that the error rates of the two models are identical, suggesting that there is no significant difference in the performance between the two models.

### Risk factor analysis

Through the individual sensitivity analysis, we observed that modifying each selected PDHI within its observed range uniquely influenced the model's predictions for specific patients (Fig. 2a–g). This highlighted the distinct impact each risk factor had on the predicted outcome. For instance, as the value of SIRI increased, the probability of predicting a positive outcome for samples that were originally negative also increased. The RDW_CV displayed a notable trend: as the value increased, samples that were originally negative initially saw an increased probability of being predicted as positive, followed by a decrease. The trends for other indicators were more complex, with the probability variation for individual samples demonstrating polymorphism, likely due to intricate interactions. This indicates the existence of complex interactions leading to diverse trends in single sample probability variations.

Our global sensitivity analysis revealed that among the independent predictive factors, SIRI exerted the most significant influence on the predictive outcome, with a value of 0.117 (Fig. 2h). This value represents the degree of change in the model's predicted outcome when SIRI values are shuffled, thereby disrupting their correlation with the target variable. The second most influential factor was HCT, with a value of 0.108. All other examined factors exhibited values less than 0.08, indicating a lesser degree of influence on the prediction outcome. This suggests that, in the context of forecasting acute ischemic stroke occurrence in elderly hypertensive patients with TIA, the impact of a single PDHI appears relatively limited. In parallel with the global sensitivity analysis, we employed SHAP values for a comprehensive feature importance analysis (Fig. 2i). The results revealed that the top five contributors to the model, in order, were: SIRI, RDW_CV, BAS_p, HCT, and PLT. Apart from SIRI, the overall contribution rankings of factors in the model differed from those obtained in the global sensitivity analysis. These analyses highlight the intricate interplay of selected PDHIs in determining the outcome variable.

Finally, we sought to elucidate potential interaction effects within our XGB-PDHIs model by conducting a pairwise analysis of all PDHIs using SHAP interaction values. Our analysis, conducted at the individual level, revealed complex interactions between different pairs of PDHIs. For illustrative purposes, we visualized the interaction effects involving SIRI (Fig. 3). Positive SHAP interaction values imply that the synergistic presence of two features increases the risk of elderly hypertensive TIA patients subsequently developing Acute Ischemic Stroke (AIS). Conversely, negative SHAP interaction values signify that the combined existence of two features reduces the likelihood of a positive prediction, thus amplifying the probability of these patients not suffering from AIS in the future. In Fig. 3, SIRI is shown to have significant non-linear interactions with each of the selected PDHIs. For instance, Fig. 3a displays the impact of different SIRI and BAS_p values on their interaction as captured by the XGB-PDHIs model. The graph demonstrates that as SIRI values increase, the direction and strength of their interaction with BAS_p values vary within different SIRI ranges. Initially, there is an enhancement in the positive interaction when BAS_p values are low, followed by a stronger positive interaction with high BAS_p values, and then a stronger negative interaction emerges as BAS_p values remain high. Subsequently, increased negative interaction occurs when BAS_p values are low again. Overall, the interaction between these two variables transitions from positive to negative enhancement. In Fig. 3b, within the same range of SIRI values, the impact of RDW_CV values on their interaction is dichotomous: higher RDW_CV values are associated with a strong positive interaction, while lower RDW_CV values correlate with a strong negative interaction. Then, the pattern reverses, showing a strong negative interaction with high RDW_CV values, and a strong positive interaction with low RDW_CV values. Similar trends are observed with other variables interacting with SIRI, indicating a complex pattern of interactions within the components of the XGB-PDHIs model. This complexity underscores the interdependent and regulatory nature of hematological indicators within the body's internal environment.

## Discussion

A vast array of studies has employed machine learning and statistical methods for AIS prediction. However, most of these studies focus on the prognosis of AIS, while research specifically aimed at predicting the incidence of AIS is less common^37–39^. Studies focusing on AIS incidence risk frequently address AIS as a uniform condition or may introduce a single stratifying factor, such as hypertension or diabetes, to forecast AIS occurrences^11,12^. Research that incorporates multiple stratifying factors to identify specific populations, such as forecasting in elderly diabetic patients or in hypertensive patients with coronary artery disease, remains relatively uncommon^40,41^. This scarcity can largely be attributed to the challenges in gathering large sample sizes for specific populations defined by numerous restrictive criteria. Furthermore, when multiple criteria are used to define a study population, the complexity of interactions among variables often increases and becomes more intricate. Traditional statistical models often fall short in accurately analyzing these intricate interactions, thereby limiting our understanding of AIS risk factors in these targeted cohorts. Our study overcomes these issues by extracting data from the HIS of a national-level neuro-specialty hospital, thereby ensuring a substantial sample size. We employed the XGBoost model to fully utilize the non-linear interactions between input variables^42,43^. Innovatively, we predicted the occurrence of AIS within three years in a patient cohort defined by three stratifying factors: elderly age, transient ischemic attack (TIA), and hypertension. Each of these is a key factor for AIS incidence^4,9,44^, and older hypertensive patients with TIA are undoubtedly a high-risk group in need of predictive assessment for AIS. We opted for the simplest model comprising only seven PDHIs ('SIRI', 'HCT', 'RDW_CV', 'PLT', 'BAS_p', 'IG_p', and 'EOS'), given its comparable performance to more complex models. This decision was based on balancing predictive accuracy with practicality for clinical application, ensuring both efficacy and ease of use for future research and practical deployment.

Machine learning significantly enhances stroke prediction accuracy by focusing on pivotal risk factors and utilizing extensive healthcare datasets^45^. Recent reviews identified several commonly used ML algorithms in cerebrovascular risk assessment, such as support vector machines, artificial neural networks, linear and logistic regression, and tree-based methods like random forests and gradient tree boosting^45–47^. Due to the lack of models specifically designed for predicting AIS in elderly hypertensives with TIA, we screened 15 models incorporating these algorithms. XGBoost emerged as the top performer. Its advanced tree-building and regularization techniques provide nuanced pattern recognition and help mitigate overfitting, rendering it particularly adept at predicting AIS within specific patient demographics^48^. Ruixuan Huang et al., using data from the Chinese Longitudinal Healthy Longevity Study (CHADS) and similar class imbalance techniques as our study, constructed multifactorial stroke prediction models for the elderly. The performance of these models was as follows: Logistic Regression (Recall: 0.75, Specificity: 0.68, AUC: 0.72), SVM (Recall: 0.70, Specificity: 0.72, AUC: 0.71), and Random Forest (Recall: 0.62, Specificity: 0.79, AUC: 0.71)^49^. Yuexin Qiu et al. compared multiple tree-based models after hyperparameter tuning in a large sample study of 46,240, finding the best performances in random forest (sensitivity: 0.778, specificity: 0.913, AUC: 0.924) and XGBoost (sensitivity: 0.776, specificity: 0.916, AUC: 0.924)^50^. Chuan Hong et al., using neural networks and random survival forests on data from diverse large-scale studies in Western populations, fitted models for subgroups based on race, sex, and age, with the highest AUC for neural networks at 0.75 and for random survival forests at 0.73^51^. Our XGB-PDHIs model (Sensitivity: 0.869, Specificity: 0.936, AUC: 0.970) not only surpasses the performance of the above-mentioned specific cohort models but is also precisely tailored for a more narrowly defined specific high-risk population: elderly hypertensive patients with TIA. The input variables for this model, derived from easily accessible clinical laboratory data, enhance its practicality and suitability for clinical application.

Our analysis prominently identifies SIRI as the most significant predictive factor, a consistent finding across global sensitivity and feature importance analyses, reaffirming its pivotal role in our model. SIRI, indicative of systemic immune-inflammation, is calculated from neutrophil, monocyte, and lymphocyte counts, and is integral in reflecting the balance between inflammatory and immune responses^52^. Parameters like HCT, RDW_CV, and PLT, linked to erythrocyte and platelet series, have been widely acknowledged in numerous studies for their association with AIS development and progression^24,53,54^. These factors, relating to blood's oxygen-carrying capacity, erythrocyte size variability, and clotting potential, are fundamentally connected to AIS via pathways like inflammation, oxidative stress, endothelial dysfunction, hemostatic balance and regulation of coagulation mechanism^20,24,53–55^. While BAS_p, IG_p, and EOS in AIS have been less explored, their potential in providing unique predictive insights cannot be overlooked. A study indicates that eosinophil cationic protein, a marker of eosinophil activity and degranulation, when elevated, is associated with an increased incidence of AIS^56^. IG has been recommended as a new indicator of systemic inflammation, showing potential to predict AIS risk^57^. There has also been a report of BAS being successfully used as one of the input variables in machine learning to predict AIS^58^. Notably, apart from SIRI's consistent top ranking, the order of other indicators varies in global sensitivity and SHAP value-based feature importance analyses. As global sensitivity analysis evaluates the impact of individual input variability on predictions, SHAP values provide insights into both the direct and interaction effects of features on model outputs. Such differential ranking highlights the complex nature of vascular mechanisms in the pathogenesis of AIS, where each predictor's biological significance may vary depending on interactions with other factors^59^. Utilizing SHAP interaction value plots, our study has uncovered, for the first time, the intricate and non-linear interplay among various hematological indicators (SIRI, HCT, RDW_CV, PLT, IG_p, BAS_p and EOS) in elderly hypertensive patients with TIA. We observed that these interactions exhibit considerable complexity and demonstrate varying trends across individuals, depending on the values of different hematological indicators, underscoring the necessity for personalized risk prediction for AIS within this demographic. Our XGB-PDHIs model emerges as a promising tool for such individualized predictions.

### Advantage and limitation

Our study introduces a precise XGBoost model, meticulously developed to predict AIS progression within three years in elderly hypertensive patients with TIA. This model utilizes a rigorous workflow and focuses on key PDHIs. We conducted an in-depth analysis of the non-linear interactions between these PDHIs, elucidating their collective impact at an individual level in the assessment of AIS risk within this demographic. Our study also has some limitations. First, our findings were derived from a single-center dataset, which may limit the generalizability of our results. Multi-center studies with diverse patient cohorts would be beneficial in validating and refining our predictive model. Second, our analysis was primarily centered on the pairwise interactions among variables. The investigation into more complex interactions involving more than two factors, as well as the establishment of thresholds for interaction effects, remains unexplored. These elements are key areas for our future research efforts. Third, although our XGBoost model shows promising results, machine learning offers possibilities for further improvement. Future research could explore alternative models and reassess feature importance to potentially enhance our findings. Last, we recognize the potential influence of additional factors such as nutrition, socioeconomic, and psychosocial elements on the onset of AIS. Integrating these factors into our analysis could improve the predictive accuracy and offer a more comprehensive understanding of AIS risk in elderly hypertensive patients with TIA.

## Conclusion

We developed an optimized XGBoost model using selected PDHIs (XGB-PDHIs), which performed competitively against more complex models incorporating a wider range of variables. This indicates the efficacy of the XGB-PDHIs in capturing the primary key variations necessary for accurate AIS prediction over a three-year period in elderly hypertensive patients with TIA. Through model interpretability analysis and SHAP interaction value plots, our study revealed the importance of nonlinear interactions among SIRI, HCT, RDW_CV, PLT, BAS_p, IG_p, and EOS in assessing AIS risk within this demographic. The XGB-PDHIs model, notable for its robust performance and practicality, provides a valuable contribution to predicting AIS risk by enabling more targeted screening and personalized risk assessment. Future work should focus on validating these findings in larger, multicenter studies and further investigating the interaction mechanisms that link key PDHIs to AIS risk.

### Supplementary Information

Supplementary Information 1.

Supplementary Information 2.

Supplementary Figures.

Supplementary Table S1.

Supplementary Table S2.

Supplementary Table S3.

## Data Availability

Due to data security reasons, the Huanhu data derived from the hospital's systems are not publicly available, but can be obtained from the corresponding author upon reasonable request for research purposes. The data will be updated and supplemented in real time.

## Code availability

The code used for feature selection, model hyperparameter selection, and calibration will be made available at https://github.com/nkchangshu/PDHIs_ML/tree/main.

## References

[1] Easton, J. D. *et al.* Definition and evaluation of transient ischemic attack: a scientific statement for healthcare professionals from the American Heart Association/American Stroke Association Stroke Council; Council on Cardiovascular Surgery and Anesthesia; Council on Cardiovascular Radiology and Intervention; Council on Cardiovascular Nursing; and the Interdisciplinary Council on Peripheral Vascular Disease. The American Academy of Neurology affirms the value of this statement as an educational tool for neurologists. *Stroke* **40**, 2276–2293. 10.1161/STROKEAHA.108.192218 (2009). 10.1161/STROKEAHA.108.192218 19423857

[2] Panuganti, K. K., Tadi, P. & Lui, F. in *StatPearls* (2023).

[3] Johnston SC, Gress DR, Browner WS, Sidney S (2000). Short-term prognosis after emergency department diagnosis of TIA. JAMA.

[4] Ghozy S (2021). Transient ischemic attacks preceding ischemic stroke and the possible preconditioning of the human brain: A systematic review and meta-analysis. Front. Neurol..

[5] Johnston DC, Hill MD (2004). The patient with transient cerebral ischemia: A golden opportunity for stroke prevention. CMAJ.

[6] Sadighi A (2019). Six-month outcome of transient ischemic attack and its mimics. Front. Neurol..

[7] Kernan WN (2014). Guidelines for the prevention of stroke in patients with stroke and transient ischemic attack: A guideline for healthcare professionals from the American Heart Association/American Stroke Association. Stroke.

[8] Kleindorfer DO (2021). 2021 Guideline for the prevention of stroke in patients with stroke and transient ischemic attack: A guideline from the American Heart Association/American Stroke Association. Stroke.

[9] Turin TC (2016). Hypertension and lifetime risk of stroke. J. Hypertens..

[10] Kaur M, Sakhare SR, Wanjale K, Akter F (2022). Early stroke prediction methods for prevention of strokes. Behav. Neurol..

[11] Chang HW (2023). Ischemic stroke prediction using machine learning in elderly Chinese population: The Rugao Longitudinal Ageing study. Brain Behav..

[12] Shao X (2023). Development and validation of risk prediction models for stroke and mortality among patients with type 2 diabetes in northern China. J. Endocrinol. Invest..

[13] Gong P (2021). The association of neutrophil to lymphocyte ratio, platelet to lymphocyte ratio, and lymphocyte to monocyte ratio with post-thrombolysis early neurological outcomes in patients with acute ischemic stroke. J. Neuroinflamm..

[14] Feng GH, Li HP, Li QL, Fu Y, Huang RB (2017). Red blood cell distribution width and ischaemic stroke. Stroke Vasc. Neurol..

[15] McCabe DJ (2004). Platelet degranulation and monocyte-platelet complex formation are increased in the acute and convalescent phases after ischaemic stroke or transient ischaemic attack. Br. J. Haematol..

[16] Siedlinski M (2020). White blood cells and blood pressure: A Mendelian randomization study. Circulation.

[17] Zhang Y, Xing Z, Zhou K, Jiang S (2021). The predictive role of Systemic Inflammation Response Index (SIRI) in the prognosis of stroke patients. Clin. Interv. Aging.

[18] Jhuang YH (2019). Neutrophil to lymphocyte ratio as predictor for incident hypertension: A 9-year cohort study in Taiwan. Hypertens. Res..

[19] Chan KL (2021). Elevated neutrophil to lymphocyte ratio associated with increased risk of recurrent vascular events in older minor stroke or TIA patients. Front. Aging Neurosci..

[20] Kellert L (2011). Cerebral oxygen transport failure?: Decreasing hemoglobin and hematocrit levels after ischemic stroke predict poor outcome and mortality: STroke: RelevAnt Impact of hemoGlobin, Hematocrit and Transfusion (STRAIGHT)–an observational study. Stroke.

[21] Emamian M (2017). Association of hematocrit with blood pressure and hypertension. J. Clin. Lab Anal..

[22] Palm F (2013). Stroke seasonality associations with subtype, etiology and laboratory results in the Ludwigshafen Stroke Study (LuSSt). Eur. J. Epidemiol..

[23] Seo SG (2020). The association between red cell distribution width and incident hypertension in Korean adults. Hypertens. Res..

[24] Xie KH (2022). Red cell distribution width: A novel predictive biomarker for stroke risk after transient ischaemic attack. Ann. Med..

[25] Yoon YZ, Kotar J, Yoon G, Cicuta P (2008). The nonlinear mechanical response of the red blood cell. Phys. Biol..

[26] Gregorich M, Strohmaier S, Dunkler D, Heinze G (2021). Regression with highly correlated predictors: Variable omission is not the solution. Int. J. Environ. Res. Public Health.

[27] Strutt JPB (2023). Machine learning-based detection of adventitious microbes in T-cell therapy cultures using long-read sequencing. Microbiol. Spectr..

[28] Lai JP (2023). Tree-based machine learning models with Optuna in predicting impedance values for circuit analysis. Micromachines.

[29] Wei TT (2023). Development and validation of a machine learning model for differential diagnosis of malignant pleural effusion using routine laboratory data. Ther. Adv. Respir. Dis..

[30] Liu M (2023). A computational framework of routine test data for the cost-effective chronic disease prediction. Brief Bioinform.

[31] Rafiei A, Ghiasi Rad M, Sikora A, Kamaleswaran R (2023). Improving mixed-integer temporal modeling by generating synthetic data using conditional generative adversarial networks: A case study of fluid overload prediction in the intensive care unit. Comput. Biol. Med..

[32] Gozukara Bag HG (2023). Estimation of obesity levels through the proposed predictive approach based on physical activity and nutritional habits. Diagnostics.

[33] Chen TL (2021). Domain specific word embeddings for natural language processing in radiology. J. Biomed. Inform..

[34] Mei X (2020). Artificial intelligence-enabled rapid diagnosis of patients with COVID-19. Nat. Med..

[35] Hammoud B (2023). Predicting incomplete occlusion of intracranial aneurysms treated with flow diverters using machine learning models. J. Neurosurg..

[36] Riley RD (2020). Calculating the sample size required for developing a clinical prediction model. BMJ.

[37] Pacchiano F (2023). Artificial intelligence applied in acute ischemic stroke: From child to elderly. Radiol. Med..

[38] Yang Y (2023). The predictive performance of artificial intelligence on the outcome of stroke: A systematic review and meta-analysis. Front. Neurosci..

[39] Liu Y, Luo Y, Naidech AM (2023). Big data in stroke: How to use big data to make the next management decision. Neurotherapeutics.

[40] Zheng X, Fang F, Nong W, Feng D, Yang Y (2021). Development and validation of a model to estimate the risk of acute ischemic stroke in geriatric patients with primary hypertension. BMC Geriatr..

[41] Coca A (2008). Predicting stroke risk in hypertensive patients with coronary artery disease: A report from the INVEST. Stroke.

[42] Khajehpiri B (2022). Survival analysis in cognitively normal subjects and in patients with mild cognitive impairment using a proportional hazards model with extreme gradient boosting regression. J. Alzheimers Dis..

[43] Zuranski AM, Gandhi SS, Doyle AG (2023). A machine learning approach to model interaction effects: Development and application to alcohol deoxyfluorination. J. Am. Chem. Soc..

[44] Ma Q (2021). Temporal trend and attributable risk factors of stroke burden in China, 1990–2019: An analysis for the Global Burden of Disease Study 2019. Lancet Public Health.

[45] Daidone M, Ferrantelli S, Tuttolomondo A (2024). Machine learning applications in stroke medicine: Advancements, challenges, and future prospectives. Neural Regen. Res..

[46] Heo J (2019). Machine learning-based model for prediction of outcomes in acute stroke. Stroke.

[47] Boyd C (2021). Machine learning quantitation of cardiovascular and cerebrovascular disease: A systematic review of clinical applications. Diagnostics.

[48] Qinghe Z, Wen X, Boyan H, Jong W, Junlong F (2022). Optimised extreme gradient boosting model for short term electric load demand forecasting of regional grid system. Sci. Rep..

[49] Wu Y, Fang Y (2020). Stroke prediction with machine learning methods among older Chinese. Int. J. Environ. Res. Public Health.

[50] Qiu Y (2023). Development of rapid and effective risk prediction models for stroke in the Chinese population: A cross-sectional study. BMJ Open.

[51] Hong C (2023). Predictive accuracy of stroke risk prediction models across black and white race, sex, and age groups. JAMA.

[52] Xia Y (2023). Systemic Immune Inflammation Index (SII), System Inflammation Response Index (SIRI) and risk of all-cause mortality and cardiovascular mortality: A 20-year follow-up cohort study of 42,875 US adults. J. Clin. Med..

[53] Sico JJ (2018). Association between admission haematocrit and mortality among men with acute ischaemic stroke. Stroke Vasc. Neurol..

[54] Franks ZG, Campbell RA, Weyrich AS, Rondina MT (2010). Platelet-leukocyte interactions link inflammatory and thromboembolic events in ischemic stroke. Ann. N. Y. Acad. Sci..

[55] Liu Y (2020). Combined prognostic significance of D-dimer level and platelet count in acute ischemic stroke. Thromb. Res..

[56] Sundstrom J (2017). Eosinophil cationic protein, carotid plaque, and incidence of stroke. Stroke.

[57] Korkut M, Selvi F, Bedel C (2022). Echocardiographic epicardial fat thickness and immature granulocyte are novel inflammatory predictors of acute ischemic stroke: A prospective study. Sao Paulo Med. J..

[58] O'Connell GC (2022). Use of deep artificial neural networks to identify stroke during triage via subtle changes in circulating cell counts. BMC Neurol..

[59] Sierra C, Coca A, Schiffrin EL (2011). Vascular mechanisms in the pathogenesis of stroke. Curr. Hypertens. Rep..

