# Supplementary material for: Acute ischemic stroke prediction and predictive factors analysis using hematological indicators in elderly hypertensives post-transient ischemic attack
Source: Sci Rep. 2024 Jan 6;14:695. doi: 10.1038/s41598-024-51402-2 (PMC10771433; doi:10.1038/s41598-024-51402-2)
Supplement: Supplementary file 1 — Supplementary Information 1. [file 41598_2024_51402_MOESM1_ESM.docx]

**Introduction to routine blood test and PDHIs**

**1. Introduction to the routine blood test and PHIs described in the paper**

At Tianjin Huanhu Hospital, the routine blood test (RBT), also known as the complete blood count or routine complete blood count, is performed using the Sysmex XE 5000 Hematology Analyzer. The RBT results are presented to clinicians in paper reports for the diagnosis and treatment of clinical diseases (see Figure 1). All RBT data is stored in the hospital's library information system (LIS), and can be exported in the form of a SQL language table by building an intermediate library. The following table shows the Chinese and English names and abbreviations of the indicators included in a patient's routine blood test report. Routine blood test, examining various cellular components, yield critical Primary Hematological Indicators (PHIs) that reflect a patient's steady-state physiological condition. These markers remain relatively stable unless influenced by specific conditions such as hematological disorders or acute infections. Thus, they provide valuable baseline information about a patient's health status. Derived Hematological Indicators (DHIs), calculated from these primary measures through various mathematical manipulations, further enrich our understanding of the patient's condition. Collectively referred to as Primary and Derived Hematological Indicators (PDHIs), these measures can deliver a comprehensive snapshot of the patient's hemodynamic status.

| In our study, we utilized a total of 28 PHIs that correspond to those found in a routine blood test report. | | |
| --- | --- | --- |
| Chinese name | English name | Short name |
| 中性粒细胞绝对值 | absolute neutrophil count | NEU |
| 中性粒细胞百分比 | percentage of neutrophils among the white blood cells | NEU_p |
| 单核细胞绝对值 | absolute monocyte count | MON |
| 单核细胞百分比 | percentage of monocytes among the white blood cells | MON_p |
| 嗜酸性粒细胞绝对值 | absolute eosinophil count | EOS |
| 嗜酸性粒细胞百分比 | percentage of eosinophils among the white blood cells | EOS_p |
| 嗜碱性粒细胞绝对值 | absolute basophil count | BAS |
| 嗜碱性粒细胞百分比 | percentage of basophils among the white blood cells | BAS_p |
| 红细胞分布宽度SD | standard deviation of the red cell distribution width | RDW-SD |
| 红细胞分布宽度CV | coefficient of variation of the red cell distribution width | RDW-CV |
| 大型血小板比率 | platelet-large cell ratio | P_LCR |
| 幼稚中性粒细胞绝对值 | absolute immature granulocyte count | IG |
| 幼稚中性粒细胞百分比 | percentage of immature granulocytes among the white blood cells | IG_p |
| 成熟中性粒细胞绝对值 | absolute mature neutrophil count | NEUT |
| 成熟中性粒细胞百分比 | percentage of mature neutrophils among the white blood cells | NEUT_p |
| 血小板压积 | plateletcrit | PCT |
| 血小板分布宽度 | platelet distribution width | PDW |
| 平均血小板体积 | mean platelet volume | MPV |
| 血小板计数 | platelet count | PLT |
| 平均血红蛋白浓度 | mean corpuscular hemoglobin concentration | MCHC |
| 平均血红蛋白含量 | mean corpuscular hemoglobin | MCH |
| 红细胞平均体积 | mean corpuscular volume | MCV |
| 红细胞压积 | hematocrit | HCT |
| 血红蛋白 | hemoglobin | HGB |
| 红细胞计数 | red blood cell count | RBC |
| 淋巴细胞绝对值 | absolute lymphocyte count | LYM |
| 淋巴细胞百分比 | percentage of lymphocytes among the white cells | LYM_p |
| 白细胞计数 | white blood cell count | WBC |
| 有核红细胞绝对值 | absolute nucleated red blood cell count | NRBC |
| 有核红细胞百分比 | percentage of nucleated red blood cells among the red cells | NRBC_p |


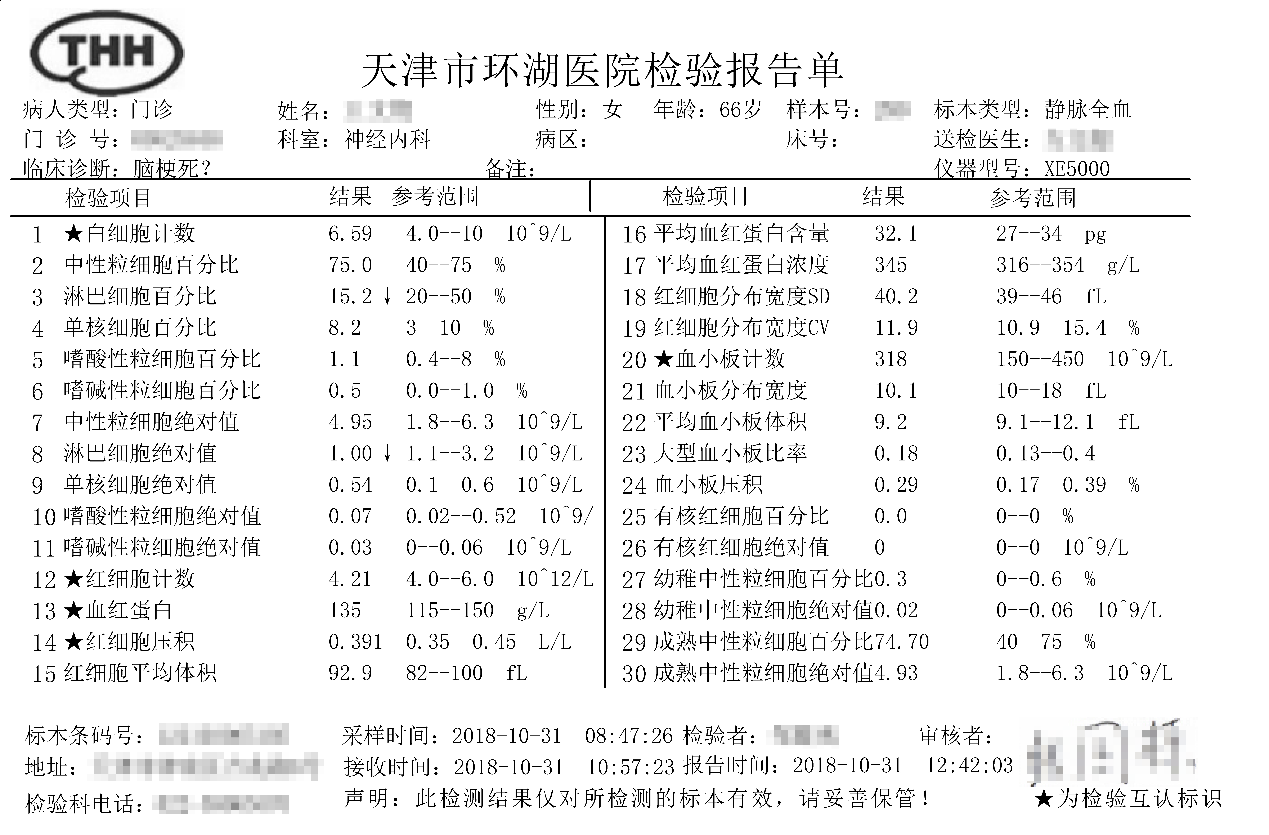


Figure 1 Example patient routine blood report from the hematology analyzer (Sysmex XE 5000) in Tianjin Huanhu Hospital.

**2. Introduction to the calculated DHIs described in the paper**

Through a comprehensive literature review, we have identified the calculated DHIs that have been independently associated with the occurrence, development, and prognosis of ischemic stroke. These variables are obtained by performing feature engineering techniques, such as mathematical operations, on the raw RBT indicators. Given their continuous nature and promising potential as independent predictors of ischemic stroke, these variables have gained considerable interest in clinical research. The following table displays the names and calculation methods of all the calculated DHIs used in the study.

| In our study, we utilized a total of 8 DHIs listed below | | | |
| --- | --- | --- | --- |
| abbreviation | full name | calculation method | reference PMID |
| NLR | neutrophil to lymphocyte Ratio | NEU/LYM | 28829826 |
| LMR | lymphocyte to monocyte ratio | LYM/MON | 28847530 |
| PWR | platelet to white blood cell ratio | PLT/WBC | 27372596 |
| PNR | platelet to neutrophil ratio | PLT/NEU | 30737090 |
| PLR | platelet to lymphocyte ratio | PLT/LYM | 30578944 |
| SIII | systemic immune inflammation index | (PLT*NEU)/LYM | 35780134 |
| SIRI | systemic inflammation response index | (NEU*MON)/LYM | 35588444 |
| RCI | red cell index | (RBC*HGB)/(LYM*PLT) | 33943024 |
