# Supplementary material for: Acute ischemic stroke prediction and predictive factors analysis using hematological indicators in elderly hypertensives post-transient ischemic attack
Source: Sci Rep. 2024 Jan 6;14:695. doi: 10.1038/s41598-024-51402-2 (PMC10771433; doi:10.1038/s41598-024-51402-2)
Supplement: Supplementary file 2 — Supplementary Information 2. [file 41598_2024_51402_MOESM2_ESM.docx]

**Detailed Introduction to Model Evaluation Metrics**

**The evaluation metrics used for the models**

The mathematical formulas and model evaluation metrics used in this study are well-established in the literature and can be found in standard machine learning textbooks and scientific publications. To save space and avoid disrupting the flow of the text, we have not included these formulas in the main body of the manuscript. However, we provide links to relevant resources and publications in the supplementary materials for interested readers. Instead, in the main body of our research, we focus on the novel contributions and insights.

| Metrics to evaluate the models in the paper | |
| --- | --- |
| balanced_accuracy (Bal-ACC) | https://scikit-learn.org/stable/modules/generated/sklearn.metrics.balanced_accuracy_score.html |
| area under the receiver operating characteristic curve (ROC-AUC) | https://scikit-learn.org/stable/modules/generated/sklearn.metrics.roc_auc_score.html |
| area under the precision-recall curve (PR-AUC) | https://scikit-learn.org/stable/modules/generated/sklearn.metrics.average_precision_score.html |
| recall_score (Recall) | https://scikit-learn.org/stable/modules/generated/sklearn.metrics.recall_score.html |
| precision_score (Precision) | https://scikit-learn.org/stable/modules/generated/sklearn.metrics.precision_score.html |
| F1-score | https://scikit-learn.org/stable/modules/generated/sklearn.metrics.f1_score.html |
| F2-score | https://scikit-learn.org/stable/modules/generated/sklearn.metrics.fbeta_score.html |
| Cohen’s kappa score (C-kappa) | https://scikit-learn.org/stable/modules/generated/sklearn.metrics.cohen_kappa_score.html |
| Jaccard similarity coefficient score (Jaccard) | https://scikit-learn.org/stable/modules/generated/sklearn.metrics.jaccard_score.html |
| Matthews correlation coefficient (MCC) | https://scikit-learn.org/stable/modules/generated/sklearn.metrics.matthews_corrcoef.html |

Note: The content within the parentheses represents the abbreviations of various model evaluation metrics used in the article.
