# Supplementary material for: Acute ischemic stroke prediction and predictive factors analysis using hematological indicators in elderly hypertensives post-transient ischemic attack
Source: Sci Rep. 2024 Jan 6;14:695. doi: 10.1038/s41598-024-51402-2 (PMC10771433; doi:10.1038/s41598-024-51402-2)
Supplement: Supplementary file 3 — Supplementary Figures. [file 41598_2024_51402_MOESM3_ESM.docx]

**Supplemental figures**


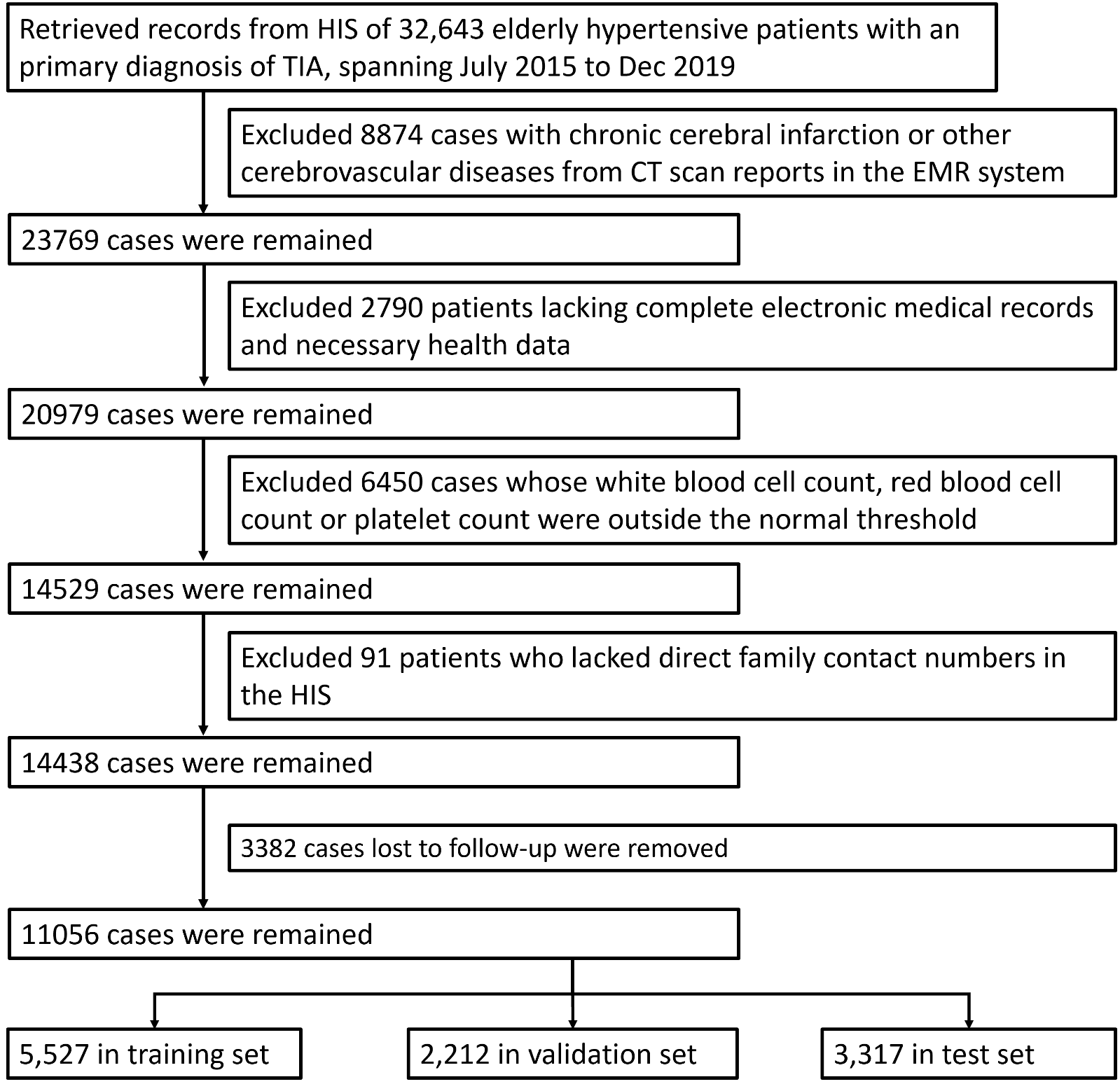


Supplemental Fig. 1 the flowchart of patient selection. This flowchart illustrates the patient selection process for the AIS prediction model. Starting with the initial cohort, it details each step of inclusion and exclusion, resulting in the final division of patients into training, validation, and test sets. Abbreviation: AIS, acute ischemic stroke; HIS, hospital information system; TIA, transient ischemic attack; EMR, electronic medical record.


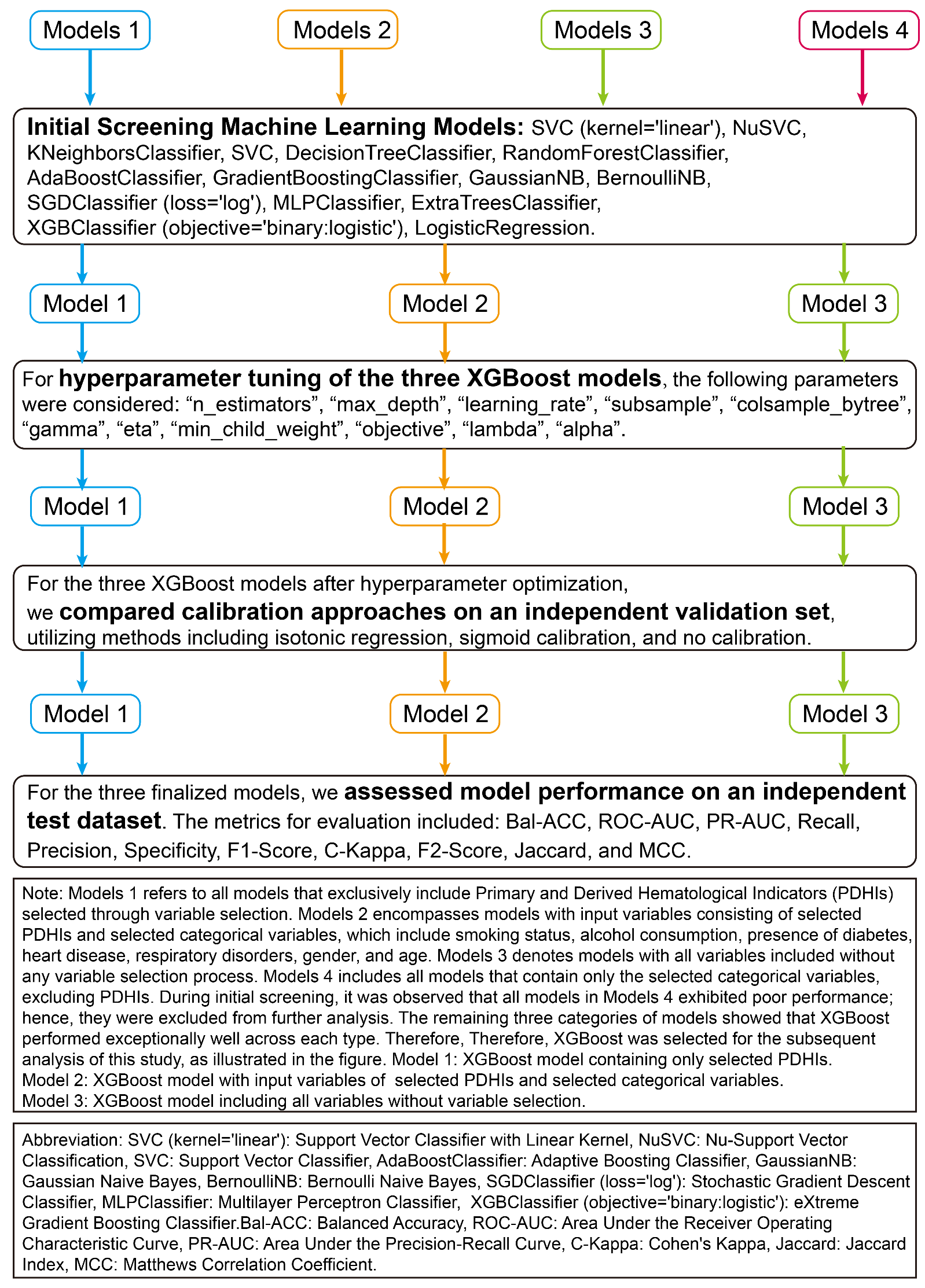


Supplemental Fig. 2 the overall workflow of our model fitting and testing


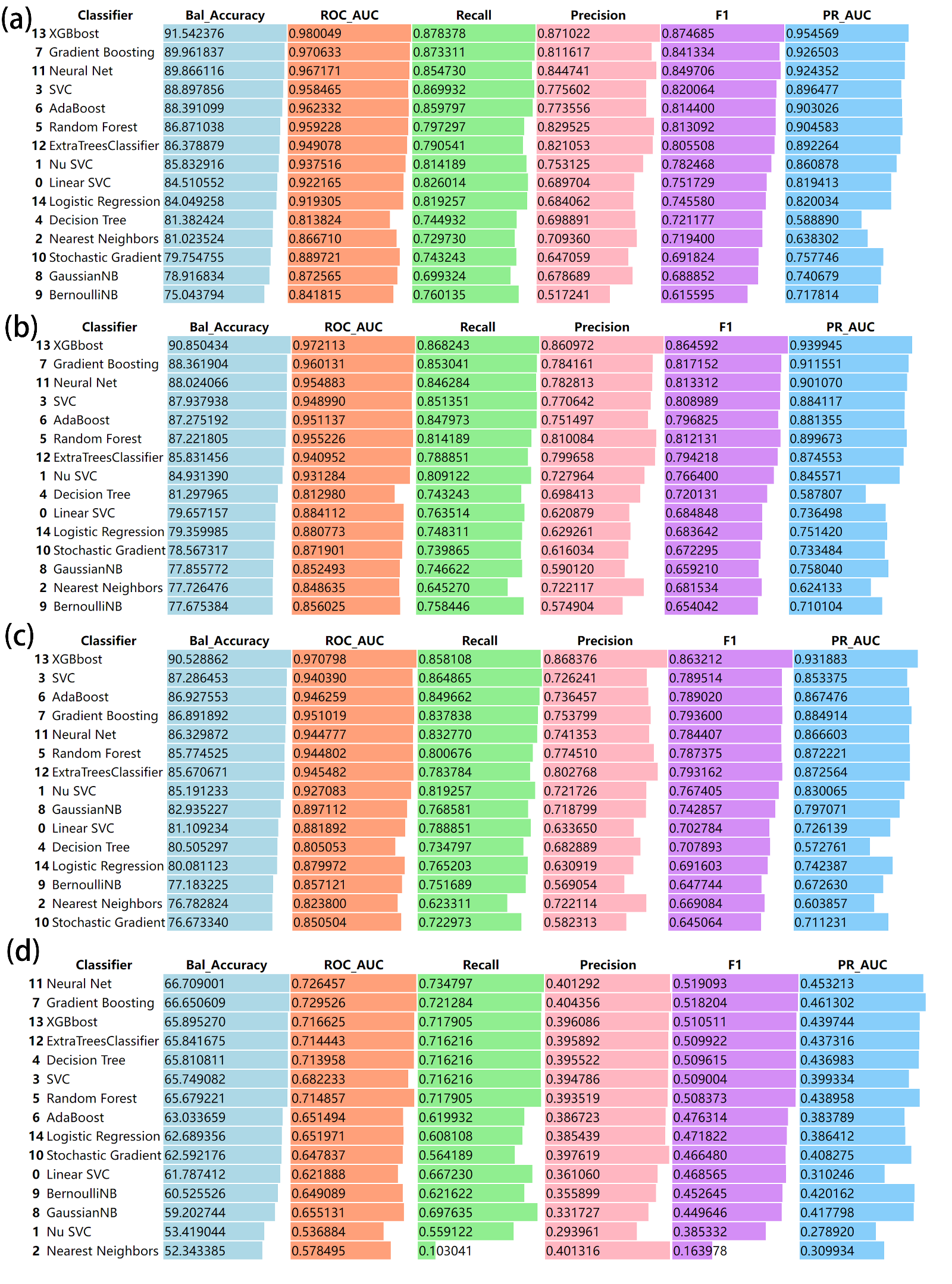


Supplemental Fig. 3: Performance comparison of initial screening machine learning models. We selected 15 machine learning models commonly utilized in literature for predicting cerebrovascular diseases. These models, fitted using the training set, were evaluated on the validation set. The prediction outcome was the occurrence or non-occurrence of an acute ischemic stroke (AIS) event. Balanced accuracy, the average of recall values for each class, represented model performance well in the context of class-imbalanced data. In our study, this served as the primary optimization metric. The models included: (a) those with all variables without any variable selection; (b) those with selected primary and derived hematological indicators (PDHIs) and categorical variables; (c) those with only selected PDHIs; and (d) those with only selected categorical variables. The selected PDHIs were SIRI, HCT, RDW_CV, PLT, BAS_p, IG_p, and EOS, and the categorical variables were smoking status, alcohol consumption, diabetes, heart disease, respiratory disorders, gender, and age. XGBoost demonstrated superior performance across models a, b, and c. For model d, which contained only selected categorical variables, all algorithms performed suboptimally. Consequently, we fitted three XGBoost models in parallel: models a, b, and c. Abbreviations: Linear SVC: Support Vector Classifier with Linear Kernel, Nu SVC: Nu-Support Vector Classification, SVC: Support Vector Classifier, AdaBoost: Adaptive Boosting Classifier, GaussianNB: Gaussian Naive Bayes, BernoulliNB: Bernoulli Naive Bayes, XGBClassifier: eXtreme Gradient Boosting Classifier, ROC-AUC: Area Under the Receiver Operating Characteristic Curve, PR-AUC: Area Under the Precision-Recall Curve.
